# Neonatal cardiovascular emergencies after hospital discharge

**DOI:** 10.1186/1824-7288-40-S2-A30

**Published:** 2014-10-09

**Authors:** Nicola Pozzi, Anna Casani, Francesco Cocca, Concetta Coletta, Maria Gabriella De Luca, Gaetano Di Manso, Lidia Grappone, Alessandro Scoppa, Luigi Orfeo

**Affiliations:** 1 Neonatal Intensive Care Unit, Maternal and Child Health Department, “G. Rummo” Hospital, via dell’Angelo 1, 82100 Benevento, Italy

## 

Neonates with out-of-hospital cardiovascular emergencies get to Emergency Department or Neonatal Intensive Care Unit in a state of shock, which is a complex clinical syndrome characterized by an acute failure of the circulatory system with inadequate tissue and organ perfusion.

It has been demonstrated that early recognition and time-sensitive aggressive resuscitation of neonatal shock significantly reduce mortality and improve outcomes [1].

The update algorithm for goal-directed treatment of neonatal shock emphasizes early use of therapies directed to restore threshold heart rates, normalize blood pressure and capillary refill ≤2 seconds and subsequent intensive hemodynamic support aimed to goals of central venous oxygen saturation (ScvO2) >70% and cardiac index (CI) >3.3 L/min/m^2^[ 2].

Rapid attainment of a vascular access is pivotal for fluid resuscitation and inotrope therapy and an intraosseous needle must be early inserted especially in the case of cardiac arrest or decompensated shock [3].

Rapid crystalloid boluses of 10-20 ml/kg (over 5-10 minutes) up to 60 ml/kg in the first hour must be administered paying attention to signs of fluid overload because overaggressive therapy can be detrimental in case of cardiogenic shock. An echocardiographic analysis can help clinicians to assess the inferior vena cava diameter or collapsibility as an index of preload status [4] and to evaluate the ventricular size and contractility to identify a cardiogenic shock [5]. Prostaglandin infusion is recommended until a ductal-dependent lesion is ruled out by trained echocardiographists.

Empiric antibiotics should be administered within one hour if a sepsis is suspected [6].

Neonates with fluid refractory shock need to start and titrate a peripheral inotropic support and progressively to be added vasopressors according to the hemodynamic state.

Many methods are available for the hemodynamic monitoring after the first hour of stabilization of the neonate. A safer ultrasound-guided central venous access can easily be obtained [7] and ScvO2 be measured. The radial arterial pressure monitoring is recommended and the arterial waveform can be used to derive additional hemodynamic information. The monitoring of CI is feasible in neonates only by non-invasive methods such as doppler echocardiography or impedance cardiography [8].

Many aspects of the intensive hemodynamic support of neonatal shock resemble those of paediatric critical care medicine (PCCM) usually provided by anaesthesiologists.

**Figure 1:**
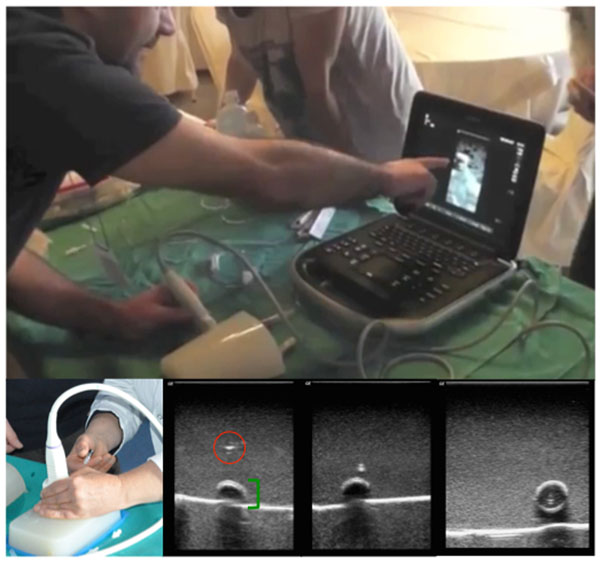
**Simulation-based training of an ultrasound-guided central venous catheterization.** The figure illustrates the ultrasound-guided vessel puncture technique by using phantoms. The trainee visualizes the simulated vessel in transverse scans (short axis) and advances the needle tip (red circle) towards the vessel (green square bracket) during the procedure.

We believe that neonatologists need a specific PCCM training before dealing with critically ill neonates and for this purpose our team has devised some courses (Figure 1) to meet these particular educational and procedural skill needs.
